# Deoxyribonucleases and Their Applications in Biomedicine

**DOI:** 10.3390/biom10071036

**Published:** 2020-07-11

**Authors:** Lucia Lauková, Barbora Konečná, Ľubica Janovičová, Barbora Vlková, Peter Celec

**Affiliations:** 1 Center for Biomedical Technology, Department for Biomedical Research, Danube University Krems, 3500 Krems, Austria; laukova.luci@gmail.com; 2 Institute of Molecular Biomedicine, Faculty of Medicine, Comenius University, 81108 Bratislava, Slovakia; basa.konecna@gmail.com (B.K.); lubica.janovicova@gmail.com (L.J.); barboravlk@gmail.com (B.V.); 3 Institute of Pathophysiology, Faculty of Medicine, Comenius University, 81108 Bratislava, Slovakia; 4 Department of Molecular Biology, Faculty of Natural Sciences, Comenius University, 81108 Bratislava, Slovakia

**Keywords:** nuclease activity, DAMPs, DNA fragmentation, inflammation, toll-like receptor

## Abstract

Extracellular DNA, also called cell-free DNA, released from dying cells or activated immune cells can be recognized by the immune system as a danger signal causing or enhancing inflammation. The cleavage of extracellular DNA is crucial for limiting the inflammatory response and maintaining homeostasis. Deoxyribonucleases (DNases) as enzymes that degrade DNA are hypothesized to play a key role in this process as a determinant of the variable concentration of extracellular DNA. DNases are divided into two families—DNase I and DNase II, according to their biochemical and biological properties as well as the tissue-specific production. Studies have shown that low DNase activity is both, a biomarker and a pathogenic factor in systemic lupus erythematosus. Interventional experiments proved that administration of exogenous DNase has beneficial effects in inflammatory diseases. Recombinant human DNase reduces mucus viscosity in lungs and is used for the treatment of patients with cystic fibrosis. This review summarizes the currently available published data about DNases, their activity as a potential biomarker and methods used for their assessment. An overview of the experiments with systemic administration of DNase is also included. Whether low-plasma DNase activity is involved in the etiopathogenesis of diseases remains unknown and needs to be elucidated.

## 1. Introduction

The presence of extracellular DNA (ecDNA) in serum was first described decades ago by Mandel and Metais [1]. DNA in the circulation originates mainly from blood and tissue cells due to apoptosis, necrosis and NETosis, but can be also produced via active secretion from living cells. The structural and genetic role of DNA as a carrier of genetic information in the nucleus completely changes after it is released into the extracellular space. The molecule of ecDNA with associated DNA-binding proteins (e.g., histones, high-mobility group binding protein 1, lactoferrin) has the ability to activate various DNA sensing receptors. These receptor systems, also known as pattern recognition receptors, are located on both sides of cell membrane and recognize ecDNA as a damage-associated molecular pattern. This activation leads to the stimulation of immune cells, induction of inflammation and NETosis [2]. Therefore, a high concentration of ecDNA in plasma was found in various inflammatory diseases and might represent a therapeutic target.

**Figure 1:**
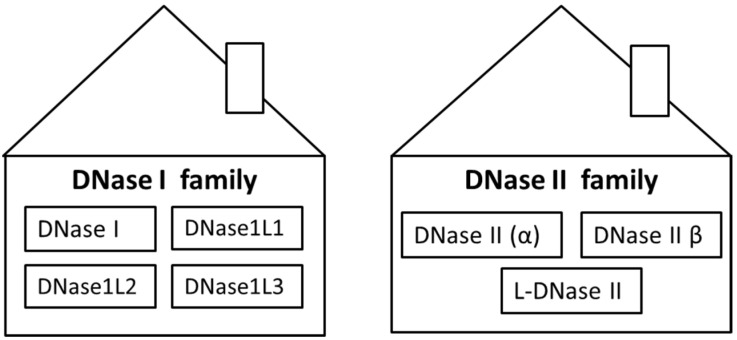
Classification of deoxyribonucleases (DNases) to two families. DNase I family includes four types of DNases: DNase I, DNase1L1, DNase1L2 and DNase1L3. DNase II family includes three types of DNases: DNase II (α), DNase II β and L-DNase II.

One of the physiological ways of how to maintain a low concentration of ecDNA is cleavage with deoxyribonucleases (DNases). DNases are enzymes which are able to hydrolyze phosphodiester bonds of DNA molecules. They can be divided into two families, which differ in biochemical and biological properties—DNase I and DNase II families (Table 1). The ability to hydrolyze DNA is common for both of them. DNases are encoded by several genes and expressed in many tissues. Some of them are secreted, and, therefore, DNases can cleave DNA in both intracellular and extracellular space. It was shown that ecDNA is generated first by cleavage inside of cells by intracellular DNases, and subsequently the fragmentation continues by extracellular DNases [3]. The evolutionary origin of DNases remains in dispute but a strongly supported hypothesis is that DNases arose in eukaryotic organisms together with phagocytosis to facilitate degradation of bacterial DNA [4]. The DNase I family includes DNase I, DNase1L1, DNase1L2 and DNase1L3, while the DNase II family consists of DNase II α and DNase II β. Although L-DNase II is considered to be part of the DNase II family, the putative gene is SERPINB1 and not DNASE as it is in the case of DNase II α and DNase II β (Figure 1). However, there are other enzymes which may not belong to a specific DNase family but are important in DNA degradation, such as three-prime repair exonucleases (TREX) TREX1 and TREX2.

## 2. DNase I

**Figure 2:**
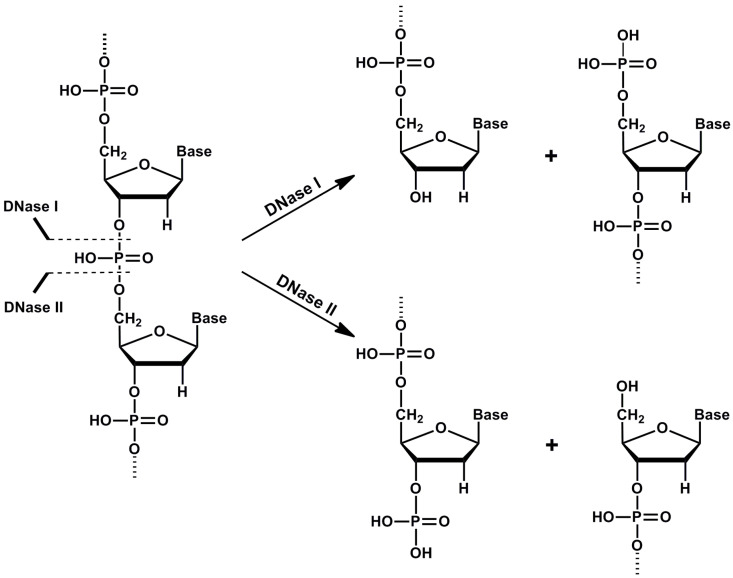
Cleavage of DNA by deoxyribonuclease I (DNase I) and deoxyribonuclease II (DNase II). DNase I cleaves DNA to form two oligonucleotide-end products with 5′-phospho and 3′-hydroxy ends, while DNase II cleaves DNA to form two oligonucleotide-end products with 5′-hydroxy and 3′-phospho ends.

DNase I, as a member of the DNase I family, cleaves DNA to form two products with 5′-phospho and 3′-hydroxy ends (Figure 2). Comparative studies showed that DNases I of vertebrate origin have a lot of characteristics in common. DNases I are active at a pH of 6.5–8 and need bivalent magnesium and calcium cations to be active [5]. Calcium ions are important for the maintenance of the optimal enzyme conformation, while magnesium ions are involved in catalysis of the cleavage of phosphodiester bonds [6]. Activity of DNase I can be effectively inhibited by addition of ion chelators such as ethylene-diamine-tetraacetic or ethylene-glycol-tetraacetic acids. Some mammalian DNases I (human, bovine, rabbit and mouse) can also be inhibited by G-actin [7]. DNase I (bovine and rat) are susceptible to inhibition by *Hypericum* extracts—especially rutin was found to inhibit activity of DNase I with potential implications on nutrition and male infertility [8]. DNase I cleaves double-stranded DNA (dsDNA) 100–500 times more effectively than single-stranded DNA (ssDNA), while structure and sequence of the DNA substrate affects the kinetics of hydrolysis [6,9]. The B-form of DNA is the most suitable substrate for DNase I compared to the Z-form, which is relatively resistant to hydrolysis [5].

DNase I is produced mainly by organs of the digestive system, such as the pancreas and salivary parotid glands. Therefore, three types of mammalian DNase I are known: pancreatic, parotid and pancreatic-parotid [10]. Evolutionary eating habits are associated with a tissue distribution of DNase I in the digestive system. Humans and pigs are omnivores and have a pancreatic type of DNase I, contrary to herbivores, such as mice and rats, which have DNase I produced in parotid glands. Bovines and rabbits produce a pancreatic-parotid type of DNase I [10]. All types of enzymes are secreted from these organs into the intestinal tract, where they are exposed to different conditions. The pancreatic type of DNase I is secreted as a component of pancreatic juice into the small intestine. This enzyme is more sensitive to pH changes than the parotid and pancreatic-parotid types of DNase I, which are able to be active even at the low pH in the stomach. It was shown that the preservation of the DNase activity of these two types of DNase I is achieved by a change of their conformation [7,11].

Human DNase I is encoded by the *DNASE1* gene located on chromosome 16 p13.3. The gene is 3.2 kb long and consists of nine exons and eight introns [12,13]. Yasuda et al. described six single-nucleotide polymorphism-based alleles of *DNASE1*-DNASE1*1–*6 [14,15]. Recently, 18 missense, 7 nonsense and 9 indel mutations in the *DNASE1* gene were described, which lead to a decreased activity or no activity of the enzyme. Some of the SNPs, such as p.Gln244Arg and p.Arg107Gly, are interspersed worldwide, and some are restricted to certain populations [16]. The polypeptide chain of DNase I contains 260 amino acid residues. Catalytic activity is ensured by four amino acid residues—Glu78, His134, Asp212 and His252 [17]. Mutational analysis revealed that amino acids Gln9, Arg41, Tyr76, Arg111, Asn170, Tyr175 and Tyr211 are also important for the activity of DNase I, because these amino acid sequences are close to the active site [18]. Structural stability of the enzyme is ensured by two Cys residues—Cys173 and Cys209—which are evolutionarily conserved in all vertebrates [19]. Another four amino acids, Glu13, Tyr65, Val67 and Ala114, are involved in G-actin-mediated inhibition of DNase I activity [20,21]. Mammalian DNases I contain two *N*-glycosylation sites—Asn18 and Asn106—which are well conserved and are both required for full enzymatic activity [22].

DNase I plays important physiological roles. DNase I, a major nuclease present in the blood and other body fluids, is responsible for the digestion of extracellular nucleoproteins, which may be crucial for the prevention of autoimmune reactions. Several studies showed that reduced DNase I activity may contribute to the development of systemic lupus erythematosus (SLE) [23,24]. Insufficient production of DNase I or the inhibition of its activity might result in an inefficient degradation of ecDNA and could be involved in the production of anti-nuclear autoantibodies (ANA) characteristic for SLE. However, Lo et al. showed that DNase I does not play the main role in degradation of plasma ecDNA alone, as no systemic change in plasma DNA size profile was observed in the knockout mouse model [25]. For better understanding of the physiology of DNase I, the *DNase1* gene was deleted in the mouse [26]. Heterozygous *DNase1^+/−^* mice and homozygous *DNase1^−/−^* were generated. Young mice of both genotypes were healthy. Later, they started to produce anti-nuclear antibodies, leading to accumulation of immune complexes in glomerular vessel walls and glomerulonephritis, as well as symptoms of SLE, and died prematurely. The prevalence of the disease phenotype was higher in females than in males, similarly to humans. Mice homozygous for the mutations developed more severe symptoms [26,27]. When DNase I deficiency was coupled with deficiency in DNase1L3 and with the induction of immune activation in the form of sterile neutrophilia, mice died due to inability to remove blood clots. This was the consequence of the fact that the neutrophils produce neutrophil extracellular traps that serve as a scaffold for thrombocytes and could not be cleaved due to deficiency in both DNases [28].

*DNase1* knockout mice were also used to determine the role of DNase I in cisplatin-induced nephrotoxicity. Cisplatin as a commonly used chemotherapeutic treatment in a wide variety of tumors has nephrotoxicity as a major side effect. Nephrotoxicity is associated with DNA fragmentation and DNase I is highly expressed in the kidney. In the experiment, it was proven that cisplatin-induced activation of DNase I might be involved in the pathogenesis. *DNase1^−/−^* mice were protected against the side effects of cisplatin treatment. This showed that the presence and activity of DNase I is crucial in the cisplatin-induced kidney injury [29].

## 3. DNase1L1, DNase1L2 and DNase1L3

Starting from 1995, the next three members of the DNase I family were described—DNase1L1, DNase1L2 and DNase1L3. These nucleases are also known as DNase I-like nucleases, because their amino acid sequences and catalytic properties are similar to DNase I [30,31]. Similar to DNase I, they are activated by Ca^2+^ and Mg^2+^ and inhibited by Zn^2+^ ions, and all of them cleave DNA to produce 5′-phospho and 3′-hydroxy ends [32]. All of these enzymes contain two important histidine amino acid residues and hydrophobic precursor peptides in the N-ends, which are conserved in all members of DNase I family. However, DNase1L2 has few unique features. While the other DNases belonging to this group are the most active under neutral pH, the maximum activity of DNase1L2 is detected in acidic pH. The second specific feature of DNase1L2 is the presence of a proline-rich domain in the center region of the protein. These different characteristics make the DNase1L2 attractive for the further research of its biological role.

The genes encoding DNase I-like nucleases are located on different chromosomes and are expressed in different organs. DNase1L3 is highly expressed in lymphoid organs [33], whereas DNase1L1 is produced in myocardium and skeletal muscles [34]. Detectable but low expression of DNase1L2 was observed in many tissues, including brain, lungs and placenta [30,32]. High expression of DNase1L2 was observed in differentiated keratinocytes of the stratum corneum, where it plays an important role in degradation of bacterial biofilms [35,36]. The secretion of DNase1L3 and DNase1L2 from these locations into the extracellular space is still unclear, whereas it has been shown that DNase1L1 is not secreted out of the cell and is localized in the cytoplasm [32,33,37]. The biological role of these enzymes is still insufficiently clarified but they may be involved in the cleavage of chromatin DNA during apoptosis [38,39]. It was demonstrated that DNase1L3 has a role in plasma ecDNA homeostasis by enhancing fragmentation, and so, in the prevention of autoimmune disorders. Concentration of DNase1L3 is elevated in ankylosing spondylitis and correlates with the clinical disease score [40]. Mice deficient in DNase1L3 have impaired DNA cleavage and the sequencing of ecDNA showed that there are alterations in the motif at the end of fragments. This finding suggests that DNase1L3 has sequence preference for DNA cleavage [41]. DNase1L3 deletion in mice was found to lead to a much higher concentration of anti-DNA antibodies when Fc gamma receptor IIB was deleted too. Double deficient mice had overactivated germinal centers which could increase the number of anti-DNA antibody-producing B cells [42]. Further studies are required for better understanding of the physiological role and evolutionary development of these enzymes.

## 4. DNase II Family

Members of this family hydrolyze the phosphodiester bonds of the DNA molecule and generate 3′-phospho and 5′-hydroxy ends (Figure 2) [43,44]. They are often called acid DNases, because they have an acidic pH optimum (pH 4.8–5.2). Increasing pH of the environment decreases their ability to cleave DNA more than 100 times [43,45]. Moreover, in comparison to DNase I family, these enzymes do not need any Ca^2+^ and Mg^2+^ cations as activators. Zinc and copper cations, sodium at high concentrations, as well as magnesium, manganese, calcium and zinc salts, strongly decrease the activity of these DNases [46,47]. The DNase II family includes two enzymes in terms of ancestry: DNase II α and DNase II β, but L-DNase II is also considered to belong to DNase II based on its properties.

## 5. DNase II α

DNase II α, also just called DNase II, is an unspecific endo-deoxyribonuclease, which cleaves phosphodiester bonds of DNA between any nucleotides, except for the terminal nucleotides at the 3′-end of the molecule [44]. The next specific feature of this enzyme is making single-strand breaks in both DNA strands; therefore, it is also called the nicking endonuclease [43,48]. Moreover, the native dsDNA is more effectively cleaved by DNase II than denaturized DNA. DNase II is expressed in most human tissues and is preferentially localized intracellularly in lysosomes. After secretion, DNase II is also present in body fluids, such as blood, saliva, urine and testicular liquid, but in relatively low amounts [49].

Human DNase II is encoded by one gene named *DNASE2,* which is located on the chromosome 19 p13.2. The sequence of *DNASE2* is 1.6 kb long and consists of six exons and five introns. Two variants of this gene are known in humans [50,51]. These two variants differ from each other in a substitution of guanine to adenine in the sequence of the promoter, resulting in reduced expression [52]. This polypeptide consists of 360 amino acids, but just a few of them are part of putative functional sites of an enzyme [53]. The hydrophobic signal peptide is located at the N-end of the enzyme, but its cleavage during posttranslational modifications is still not clarified. Seven cysteines, which are important for stability of the enzyme, are present in the amino acid sequence, whereas six of them are well conserved in mammals [54]. The polypeptide chain also contains four *N*-glycosylation sites: N86, N212, N266 and N290. These sites are important for maturation and catalytic activity of DNase II [15,55]; therefore, the presence of *N*-glycosylation inhibitors causes the decrease of its activity and the reduction of the molecular weight of the enzyme from 43 to 37 kDa [54].

DNase II α plays an important role in the metabolism and protection of the organism, related to its localization in lysosomes and the acidic pH optimum. The reaction product of DNase II cleavage carries 3′-terminal phosphate, which is the reason why this enzyme cannot be involved in replication, recombination and reparation of DNA. However, DNase II may play a role in the cleavage of chromatin DNA during apoptosis and also participates in the differentiation of red blood cells and the development of the thymus in mice [56,57]. This lysosomal enzyme also degrades the DNA of phagocytosed apoptotic bodies or the DNA entering the cell via endocytosis [58]. A recent study demonstrated that DNase II has a role in activation of the inflammatory response, because toll-like receptor 9 (TLR9) responds to DNA fragments generated by DNase II. Therefore, DNase II is required for TLR9 activation by the bacterial genomic DNA [59,60]. The significance of the physiological role of DNase II was also demonstrated by gene disruption in mice. This deficiency in homozygous *DNase II^−/−^* mice resulted in the death of mice during the later stages of the development [61,62]. This effect was caused by the constitutive production of interferon β by macrophages and definitive erythropoiesis. The next study demonstrated that chronic polyarthritis was the reason why adult DNase II-deficient mice died [63]. No link between deficiency of this enzyme and human diseases was found. However, the human DNase II polymorphisms were shown to be associated with higher risk of kidney disorder in SLE patients [64]. Future studies are required to better understand the physiological role of DNase II.

DNase II α together with DNase1L2 are two main enzymes essential for maintenance of homeostasis of skin cells. DNase II α was identified as the unexpendable enzyme in the outermost skin layer. It can play a role in making the barrier to bacteria and virus passages. DNase II α is likely transported in lamellar bodies or in sebum to the stratum corneum [65]. DNase1L2 has a negative impact on bacterial biofilm formation. Degradation of DNA in bacterial biofilms by DNase1L2 is a level of protective barrier against infection caused by skin pathogens. These findings strongly support the role of DNase1L2 as a crucial enzyme expressed in cornifying keratinocytes and sebocytes [36]. Another study described that the removal of DNA from keratinocytes during cornification is negatively affected by double deletion of both DNase II α and DNase1L2. These cells retain their DNA in nucleus-like compartments and surprisingly, this does not affect the resistance of keratinocytes to mechanic stress [35]. The production of sebum is directly linked to apoptosis of skin cells and DNase II α. Deficiency of DNase II α in differentiating sebocyte cells leads to incomplete DNA degradation and deficiency in lysosomal autophagy [66]. However, DNase1L2 was also identified as the important enzyme in the removal of DNA in cornified keratinocytes. DNase1L3 is expressed in differentiated keratinocytes, and mice tissues and human tissues differ in the expression. In the mouse model, the deficiency in DNase 1L3 makes hair less resistant to mechanical stress [67]. Finally, the double deficiency of DNase1L2 and TREX2 impacts the DNA removal of cornifying keratinocytes in tongue epithelium. The additional deficiency of TREX2 results in accumulation of DNA in cytoplasm. The accumulation of DNA in the tongue epithelium is tolerated, and with no inflammation signalization via TLR9 [68].

## 6. DNase II β

DNase II β, also called DNase II-like acid DNase, has very similar biochemical properties to DNase II α, and therefore, the catalytic activity of this enzyme is not dependent on any cofactors and maximal activity was observed under acidic conditions. This enzyme is expressed to a large extent in salivary glands, but its production was also detected in other tissues, such as trachea, lungs, testis, lymph node and prostate, from which it is secreted [69]. The gene *DNASE2B* is localized on the chromosome 1 p22.3 and consists of 6 exons. This gene is in opposite orientation to the human uricase pseudogene [70]. The protein structure of DNase II β consists of 358 amino acid residues. The sequence of protein products share 37% of the identity and 56% of the conservativeness with DNase II [70]. The first 22 amino acids of the human DNase II β are predicted to be the N-terminal signal peptide [69,71]. DNase II β also contains nine cysteine residues and four potential sites for *N*-glycosylation, which are important for the stability and full activity of this enzyme [54]. The role of the DNase II β is still not fully understood, but it was shown that it degrades nuclear DNA in the course of terminal differentiation of lens fiber cells. DNA degradation is necessary to ensure the transparency of the lens and the accumulation of undigested DNA causing cataracts [72].

## 7. L-DNase II

L-DNase II, as well as the other enzymes of this family, is active in the absence of cations and its activity is high in acidic pH. L-DNase II was prepared from porcine spleen, and after sequencing, it was shown that the peptide sequence was identical to Serpin Leukocyte Elastase Inhibitor [73]. The Leukocyte Elastase Inhibitor, as the L-DNase II precursor, is encoded by one gene named *SERPINB1*, which is located on chromosome 6q25 and contains 9 exons. The expression of this gene is ubiquitous. The native form of the Leukocyte Elastase Inhibitor protein has 42 kDa and is localized in the cytoplasm of the cell. It has an anti-protease activity and inhibits elastase, cathepsin G and proteinase 3, and thus protects tissues from the damage at inflammatory sites [74]. However, long exposure results in a decrease of the molecular weight of Leukocyte Elastase Inhibitor (35 and 27 kDa) and the loss of its anti-protease activity [73]. This form is also called L-DNase II, it migrates to the nucleus and acquires an endonuclease activity. The change of Leukocyte Elastase Inhibitor into the L-DNase II form can also be induced in vitro by Leukocyte Elastase Inhibitor incubation in an acidic pH or in the presence of different proteases, such as elastase, cathepsin G, proteinase 3 and apoptotic protease 24 [75]. The main role of L-DNase II is the participation in the DNA degradation of apoptotic cells. A recent study showed that L–DNase II is activated during photoreceptor cell death in light-induced retinal degeneration [76]. Moreover, L-DNase II is activated in differentiated cells or in tumor cells unable to activate the caspase pathway [73,77,78]. A more detailed characterization of L-DNase II in physiology and pathophysiology is needed.

## 8. TREX1 and TREX2

TREX1 is a 3′-to-5′exonuclease that is crucial in granzyme A-mediated cell death, and deficiency leads to autoimmunity [79]. This apoptosis-like cell death occurs when TREX1 binds SET complex to be exported out of the endoplasmic reticulum to cleave DNA in cytoplasm [80]. The mutation of the TREX1 gene was found in a subpopulation of SLE patients, while the variant was not observed in a population of healthy controls. The mechanism is likely based on cytosolic recognition of DNA which induces a TRL9-independent immune response [81]. The intracellular degradation of neutrophil extracellular traps is dependent on TREX1. Macrophages utilize TREX1 to remove cytosolic DNA originating from neutrophil extracellular traps. Its extracellular counterpart, DNase1L3, degrades DNA from neutrophil extracellular traps in dendritic cells [82]. TREX2 is an exonuclease that is essential in the maintenance of genome integrity of skin cells [83]. TREX2 is a homolog of TREX1, and despite the expression of TREX2 in most tissues, their role is not the same. TREX2 was shown to link transcription and mRNA export from the nucleus in mammalian cells [84].

## 9. Methods of the Measurement of DNase Activity

**Figure 3:**
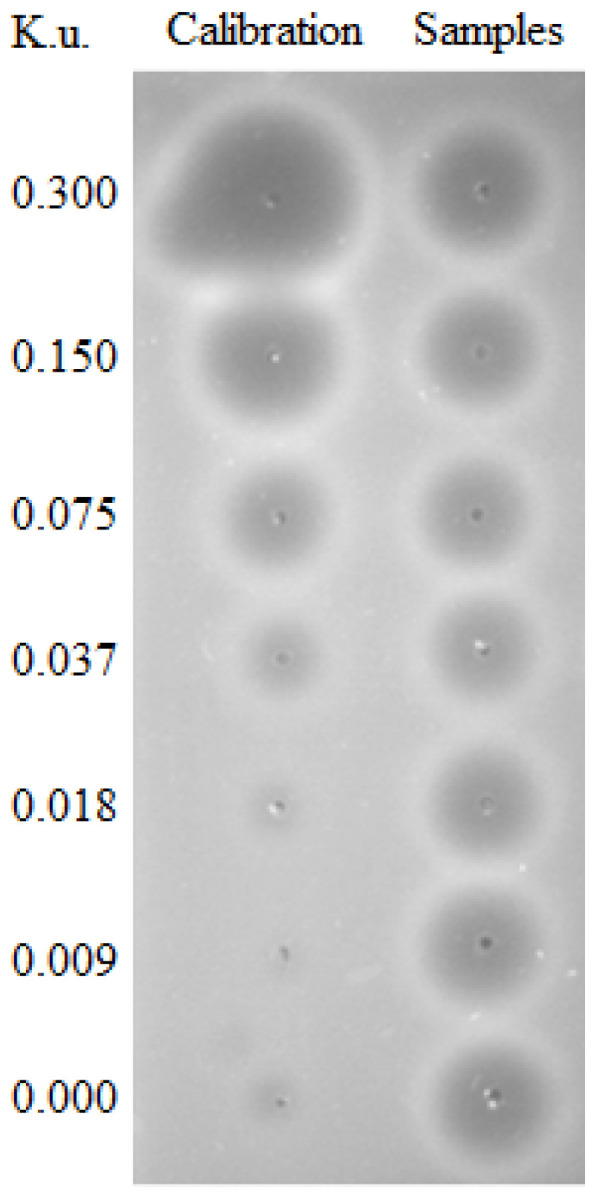
Deoxyribonuclease (DNase) activity measured using the single radial enzyme diffusion (SRED) method in Kunitz units (K.u.). Plasma samples were analyzed using a gel containing DNA after overnight incubation at 37 °C in the dark. The area of the circles represents DNA in the gel cleaved by DNase in the plasma samples. Dilutions of DNase were used as a calibrator.

In 1950, the first method of the measurement of DNase I activity was described by Kunitz [85]. He isolated and precipitated DNase from fresh beef pancreas and found that the cleavage of DNA by crystalline DNase is accompanied by the increase of absorption (λ = 260 nm) of UV light. This spectrophotometric method was then used for estimating DNase activity for the next 43 years until Nadano et al. introduced a new method for DNase I activity measurement called “single radial enzyme diffusion” (SRED, Figure 3). This simple method is based on the digestion of DNA in the agarose gel by DNase, which is present in samples punched into the gel. DNase activity is represented by the size of a dispensed circular well in an agarose gel layer, in which DNA stained by ethidium bromide is uniformly distributed. After the incubation, a circular dark zone is formed as the enzyme diffuses from the well radially into the gel and cleaves DNA (Figure 3). The diameter of the dark circle positively correlates with the amount of the enzyme applied to the well [86]. SRED underwent many modifications, which led to an increase of the sensitivity and safety, such as the replacement of ethidium bromide with SYBR Green I or other DNA gel stains [49]. The SRED method is easy and reliable, which is the reason why it is still intensively used, but it also has some limitations mainly related to reproducibility, partially caused by the small volume used. This led to efforts to introduce new methods. One of them is the kinetic colorimetric DNase I activity assay, which was originally developed for the assessment of stability of the human recombinant DNase I, also known as Pulmozyme. The method was adjusted from a colorimetric endpoint enzyme activity assay based on the degradation of a DNA/methyl green complex [87]. Another similar method is based on the ability of PicoGreen dye to enhance its fluorescence when bound to dsDNA. In this fluorometric assay, the reaction mixture of a DNase I sample and 0.2 μg of the DNA substrate is prepared in a fluorescence microtiter plate. PicoGreen reagent is added to each well at the end of the incubation and the fluorescence intensity is measured. In this case, intensity negatively correlates with DNase activity [88]. Immunochemical microtiter plate-based assays were introduced to quantify the DNase activity in plasma and urine. The principle of both assays was the cleavage of biotinylated and fluorescein-labeled PCR products of different lengths and on the immunochemical detection of non-digested DNA. The assay based on the hydrolysis of a longer (974 bp) PCR product labeled with fluorescein-labeled reverse and biotinylated forward primers was more sensitive than the other one, which is based on the hydrolysis of double-labeled 20 bp oligonucleotide [89].

The endeavors to increase the sensitivity of measurement continued, and Nakajima et al. developed the sensitive enzyme-linked immunosorbent assay (ELISA) for the measurement of DNase I in human serum. This sandwich type of ELISA measured the DNase I protein using a polyclonal antibody against this protein and a biotinylated monoclonal antibody for the detection [90]. However, it is clear that ELISA is used to quantify the protein, in this case the enzyme, but not its activity, which may be dependent on activators and inhibitors and not only on the quantity of the enzyme.

Another method for DNase activity analysis was developed two years later. It is miniaturized, rapid and needs a minimal volume of samples [91]. It was based on a microchip electrophoresis, which is a fast and sensitive assay useful for easy separation of protein and DNA. The principle of this method is endo-nucleolytical degradation of DNA by DNase I, resulting in the elimination of DNA and the reduction of intensity of fluorescence. Compared to other methods, this one is very rapid, as it can detect DNase I activity in 10 min [91]. A year later, Lee and Min developed the new simple fluorometric assay for measurement of DNA exonuclease activity based on the preferential binding of ssDNA over dsDNA to graphene oxide. The benefits of this assay are simple and the quantitative activity measurement is done in a short time at a low cost [92].

In 2015, a new method based on a lateral flow immunochemical assay for the measurement of DNase I activity was developed. The assay is based on a dually labeled dsDNA as the reporter probe. The probe has a biotin-labeled terminal bound to the streptavidin immobilized on the lateral flow test strip and the fluorescein-labeled terminal bound to the antibody-conjugated gold nanoparticles. DNase activity is measured as the test line intensity decreases, caused by the cleavage of the reporter probe [93]. In the same year, the protocol for rapid and sensitive determination of DNase activity by degradation of ethidium bromide-DNA complexes using a 96-well plate fluorescence reader was also published. As in many other methods, DNase activity is again measured by its efficiency to hydrolyze a specific amount of dsDNA in the presence of ethidium bromide in a defined time [94].

## 10. DNase Activity as a Biomarker

A high concentration of ecDNA in plasma is associated with various diseases. However, it is more than just a biomarker, because it is recognized as a damage-associated molecular pattern and activates an immune response. Clearance by DNases, mostly by DNase I, which hydrolyzes DNA in the blood, is likely one of the mechanisms that regulates the concentration of ecDNA, and in this way, prevents the induction of inflammation. So, it is not a surprise that it was shown that the DNase activity could be a biomarker of various diseases. Low DNase activity was found in patients with malignant stomach, colon and pancreas cancer, and also malignant lymphoma, compared to healthy individuals [95,96]. Conversely, high serum DNase activity was observed in patients with breast [97] and oral cancers [98]. The study of different phenotypes of DNase I showed that the phenotype 2, with DNASE1*2 polymorphism, may potentially be a good predictor of the development of gastric and colorectal carcinoma, while no such association was found with other types of cancer. The acute myocardial infarction is the next disease connected with abnormal DNase activity. Patients with ischemic heart disease and manifesting with myocardial infarction have an increased DNase activity, however this feature has not been proven as a biomarker yet [99,100,101,102]. The DNASE1*2 allele is also related to a higher risk of myocardial infarction. It could also be a genetic risk factor for the rupture of coronary atherosclerotic lesions, which leads to the myocardial infarction [103]. The role of DNase activity in biomedicine is, however, not restricted to the diagnostics or screening, ecDNA and, thus, DNase is also a key therapeutic target.

## 11. Application of DNase and DNase Treatment

DNA is a very viscous polyanion [104,105], which contributes to the high viscosity of lung secretion in cystic fibrosis and chronic obstructive pulmonary disease, both characterized by high levels of DNA in the alveoli [106]. They are released from necrotic cells [107] but also as part of the neutrophil extracellular traps [108]. Increased viscosity of the mucus is associated with a decreased mucociliary clearance. Several studies have therefore reported the use of DNase I as a possible treatment. In 1950, a group of authors showed that recombinant human DNase I decreased the viscosity of mucus in vitro within minutes [109]. Since then, the positive effects of DNase treatment were studied at the different types of respiratory system issues, such as cystic fibrosis [110], asthma, obstructive pulmonary disease and chronic pulmonary diseases (Table 2).

Several clinical studies observed the positive effect of inhaled recombinant human DNase I on the lung function of patients with severe pulmonary diseases. Therefore, for more than 20 years, recombinant human DNase I, also known as Pulmozyme or dornase alfa, is one of the most commonly used medications to treat the lung disease of cystic fibrosis. One of the benefits of the treatment with DNase I is the lack of major adverse effects, except voice alteration and rash [111]. Rarely, elastase activity can increase in sputum and induce bleeding in the murine lung [112]. But the overall positive effect of DNase I on patients with cystic fibrosis was proven repeatedly. Positive outcomes from clinical studies led to Food and Drug Administration (FDA) approval and wide use of DNase I for this treatment [113,114,115].

A similar effect of DNase was also seen in other respiratory diseases. Simpson et al. collected the pus from patients with surgically drained soft tissue abscesses and with empyema thoracis and studied the effects of streptokinase and DNase [135]. They proved that the addition of DNase allowed significantly faster passage of the pus, and later it was shown that intrapleural administration of DNase reduced pus viscosity [134]. Moreover, recombinant human DNase is considered as a potential therapy for refractory treatment of intubated patients with asthma, as confirmed by many studies [117,118,119].

SLE is a disease characterized by the production of pathogenic autoantibodies to nucleoprotein antigens, including dsDNA, and is associated with low DNase activity. This was the rationale behind the hypothesis of DNase I treatment for SLE. However, studies and experiments have provided contradictory results. One of them showed that an injection of recombinant human DNase I catalyzes the hydrolysis of ecDNA and also delays the development of dsDNA antibodies, reduces proteinuria as a marker of kidney damage and delays mortality in a lupus-prone murine model [120]. These findings were not confirmed in another experiment, in which the injection of DNase I in lupus-prone mice did not improve early or late stages of murine lupus nephritis [121]. A human study showed that recombinant human DNase I had no effect on kidney function or disease activity in patients with lupus nephritis [122]. It is not clear whether the dose and route applied was optimal, whether patient selection was ideal or whether a combination of DNases could be more effective or not. In addition, ecDNA in cystic fibrosis is in the alveoli, and so is readily available for the DNAse activity, in contrast to the cell membrane-bound chromatin in lupus nephritis that is likely protected against enzymatic degradation [136].

DNase inhibits the proliferation of tumor cells [137]. In several studies, it was shown that DNase treatment prevents blood-borne liver metastasis of transplanted subcutaneous tumor cells in mice and also ascites tumor cells in rats. The same research team showed that the intravenous administration of DNase I enhanced tumor-cell arrest in the lung microvasculature [123,124,125]. Linardou et al. tested the cytotoxic potential of mammalian DNase-I and its possible use in tumor-targeting strategies. They designed and constructed a chimeric molecule consisting of DNase I and single-chain Fv fragment antibody against the human placental alkaline phosphatase, which had both antigen-binding and DNA-cleaving activity in vitro and was highly cytotoxic in cells expressing the specific antigen [138]. DNase I thus provides a potential therapeutic strategy for targeting specific cells. However, further studies are necessary. In addition, DNase I activity could be a novel biomarker of liver cancer. While no association was observed between total ecDNA and risk of liver cancer, patients with a higher DNase activity were at risk [139].

It is known that ecDNA acts like a glue in the structural stability of biofilms in a variety of bacterial species. Since Whitchurch et al. found that DNase treatment can effectively inhibit biofilm formation [140], ecDNA became the new interesting target in biofilm control. Numerous experiments proved that DNase is effective against biofilm formation in a wide range of microbes, including *Escherichia coli* [141], *Pseudomonas aeruginosa* [140], *Haemophilus influenza* [142], *Vibrio cholera* [143], *Listeria monocytogenes* [144], *Streptococcus pneumonia* [145] and many others. Destabilization of the biofilm with DNase may increase pathogen sensitivity to antibiotics, increasing the effectiveness of the treatment.

Treatment with DNase I might eventually be helpful in neurodegenerative diseases, for example in patients with dementia in the end-stage of Alzheimer’s disease [126]. Of course, further and larger interventional studies are required in order to evaluate the role of ecDNA in the neurodegenerative pathologies. DNase I treatment is more advanced in diseases associated with inflammation caused by damage-associated molecular patterns. Treatment with DNase I improved mice survival and tissue damage in a model of hemorrhagic shock or ischemia-reperfusion injury [146]. Interestingly, one study described a positive effect of DNase I eye drops for the treatment of dry eye disease. The phase I/II clinical trial found that the eye drop treatment is safe and it reduced the symptoms by removing neutrophil extracellular traps from the ocular surface [147]. Concentration of ecDNA is one of the potentially valuable markers of systemic inflammatory response in patients after surgery or trauma. The increased ecDNA of mitochondrial origin is associated with decreased DNase activity and adverse outcomes. DNase I administration is a potential treatment targeting the immune reaction after trauma [148]. High ecDNA was also found in blood of septic patients [149,150], being a predictor of poor clinical outcome in severe sepsis [151]. Several studies confirmed the protective effect of DNase I in experimental models of sepsis. Gao et al. induced sepsis in rats by intraperitoneal injection of lipopolysaccharide. The disruption of neutrophil extracellular traps by intravenous DNase I decreased the systemic acute inflammatory response and protected the intestine from injury [127]. In another study, DNase I was administered in mice with caecal ligation and puncture modeled sepsis at two different time points. Interestingly, the delayed administration of DNase had a protective effect in sepsis, likely due to the physiological role of ecDNA in the early phases of the infection [128]. A therapeutic effect of DNase was also observed in a murine model of bacterial sepsis [130]. However, it is now unclear whether the positive effects of exogenous DNase I are mediated by the degradation of neutrophil extracellular traps, other forms of ecDNA, or by the prevention of intravascular coagulation, etc. In addition, there are differences between the studies in dose, route of administration and timing of the enzyme, which might be critical.

## 12. Conclusions

The knowledge about the biology of DNases, including the source, function and activity, is limited. It is expected that their main function is to cleave ecDNA either of the same or different origin in order to control its concentration. The variability of endogenous DNase activity is high, but the determinants of this biological variability are still unknown. In addition, the two DNase families and their subtypes should be considered separately, since it is likely that the physiology of the DNases will vary. While ecDNA is being studied in detail in various diseases, DNase as the potential ecDNA cleavage mechanism is clearly under-studied. Some studies have shown that DNase might have a broader spectrum of indications than just cystic fibrosis. Considering the therapeutic potential, it is clear that research on DNases is of importance as it might represent a crucial regulatory mechanism of inflammation and its resolution. More studies are needed to better understand the importance and possibilities of endogenous and exogenous DNase, respectively.

## Figures and Tables

**Table 1:** Characterization of different types of deoxyribonucleases (DNases).

Family	DNase I Family				DNase II Family		
Name	DNase I	DNase1L3	DNase1L1	DNase1L2	DNase II α	DNase II β	L-DNase II
Molecular mass (kDa)	38	33	34	33	43	40	27
Optimal pH	6.5–8	6.5–8	6.5–8	5.6	4.8–5.2	4.8–5.2	4.8–5.2
Activation by Mg^2+^ and Ca^2+^	+	+	+	+	-	-	-
Inhibition by EDTA/EGTA	+	+	+	+	-	-	-
Inhibition by G-actin	+	-	-	-	-	-	-
Productive organs	pancreas	spleen, liver	muscles, myocardium	brain, lungs, placenta, skin	all tissues	salivary glands	spleen
Name of coding gene	*DNASE1*	*DNASE1L3*	*DNASE1L1*	*DNASE1L2*	*DNASE2*	*DNASE2B*	*SERPINB1*
Localization of human gene	16p13.3	3p14.3	Xq28	16p13.3	19p13.2	1p22.3	6q25.2
Number of exons	17	8	10	7	6	6	9

**Table 2:** Effect of the DNase treatment in various diseases.

Disease	Organism	Administration	Dose	Effect	Reference
Cystic fibrosis	humans	inhalation	2.5 mg once or twice daily	positive	[110]
	humans	inhalation	2.5 mg/day, 1 month	positive	[116]
	mice and humans	inhalation (humans) at the nares (mice)	2.5 mg/day (humans) 50 µL of the sputum (mice)	negative	[112]
	humans	inhalation	2.5 mg/day in 2-week periods	positive	[113]
	humans	inhalation	2.5 mg/day, 28 days	positive	[114]
	humans	inhalation	2.5 mg/day, 2-times, 4 weeks, with a 4 week pause	positive	[115]
Asthma	humans	intratracheal	2.5 mg	positive	[117]
	humans	intratracheal	10 mg, 2 times 8 h apart	positive	[118]
	humans	inhalation	2.5, 5.0 or 7.5 mg	no	[119]
Systemic lupus erythematosus	mice	intraperitoneal	150 µg/day, 3 months	positive	[120]
	mice	intraperitoneal	0–15 µg/g, 1–6 months	no	[121]
	humans	single intravenous 10 subcutaneous	25 µg/kg, 125 µg/kg	no	[122]
Cancer	rats	intravenous	1.5 U	positive	[123]
	mice	intravenous	0.1 U	positive	[124]
	rats	intravenous	1 U	positive	[125]
Alzheimer disease	humans	oral	120 mg/day, 2 months	positive	[126]
	rats	intravenous	10 mg/kg	positive	[127]
Sepsis	mice	intraperitoneal	20 mg/kg (every 2, 4 or 6 h)	positive	[128]
	mice	intraperitoneal	5 mg/kg (7 times)	positive	[129]
	mice	intravenous	10 mg/kg	positive	[130]
Acute kidney injury	rats	intraperitoneal	0.1 mg/kg	positive	[131]
Acute liver injury	rats	intravenous	10 mg/kg	positive	[132]
Ischemic-reperfusion syndrome	mice	intraperitoneal intravenous	50 µg, 2 times 10 µg, 1 time	positive	[133]
Empyema thoracis	humans	intrapleural	2.5 mg (1–2 times)	positive	[134]
